# Strong shift from HCO_3_^−^ to CO_2_ uptake in *Emiliania huxleyi* with acidification: new approach unravels acclimation versus short-term pH effects

**DOI:** 10.1007/s11120-014-9984-9

**Published:** 2014-02-23

**Authors:** Dorothee M. Kottmeier, Sebastian D. Rokitta, Philippe D. Tortell, Björn Rost

**Affiliations:** 1 Alfred Wegener Institute Helmholtz Centre for Polar and Marine Research, Am Handelshafen 12, 27570 Bremerhaven, Germany; 2 Department of Earth Ocean and Atmospheric Sciences, and Deparment of Botany, University of British Columbia, Vancouver, BC V6TZ4 Canada

**Keywords:** CO_2_ concentrating mechanism, pH, Inorganic carbon source, Coccolithophore, Ocean acidification, Isotopic disequilibrium, Photosynthesis

## Abstract

Effects of ocean acidification on *Emiliania huxleyi* strain RCC 1216 (calcifying, diploid life-cycle stage) and RCC 1217 (non-calcifying, haploid life-cycle stage) were investigated by measuring growth, elemental composition, and production rates under different *p*CO_2_ levels (380 and 950 μatm). In these differently acclimated cells, the photosynthetic carbon source was assessed by a ^14^C disequilibrium assay, conducted over a range of ecologically relevant pH values (7.9–8.7). In agreement with previous studies, we observed decreased calcification and stimulated biomass production in diploid cells under high *p*CO_2_, but no CO_2_-dependent changes in biomass production for haploid cells. In both life-cycle stages, the relative contributions of CO_2_ and HCO_3_^−^ uptake depended strongly on the assay pH. At pH values ≤ 8.1, cells preferentially used CO_2_ (≥ 90 % CO_2_), whereas at pH values ≥ 8.3, cells progressively increased the fraction of HCO_3_^−^ uptake (~45 % CO_2_ at pH 8.7 in diploid cells; ~55 % CO_2_ at pH 8.5 in haploid cells). In contrast to the short-term effect of the assay pH, the *p*CO_2_ acclimation history had no significant effect on the carbon uptake behavior. A numerical sensitivity study confirmed that the pH-modification in the ^14^C disequilibrium method yields reliable results, provided that model parameters (e.g., pH, temperature) are kept within typical measurement uncertainties. Our results demonstrate a high plasticity of *E. huxleyi* to rapidly adjust carbon acquisition to the external carbon supply and/or pH, and provide an explanation for the paradoxical observation of high CO_2_ sensitivity despite the apparently high HCO_3_^−^ usage seen in previous studies.

**Electronic supplementary material:**

The online version of this article (doi:10.1007/s11120-014-9984-9) contains supplementary material, which is available to authorized users.

## Introduction

Marine phytoplankton account for ~50 % of global primary production and are the main drivers of the marine "particulate organic carbon" (POC) pump (Falkowski et al. 1998; Field et al. 1998). Calcifying phytoplankton species also contribute to the "particulate inorganic carbon" (PIC) pump and thereby play a dual role in regulating marine biogeochemical cycling of carbon through their effects on surface ocean alkalinity (Broecker and Peng 1982; Zeebe and Wolf-Gladrow 2007). One key species of calcifying phytoplankton is the cosmopolitan and bloom-forming coccolithophore *Emiliania huxleyi*, which has been established as a model organism over the recent decades (Paasche 2002; Raven and Crawfurd 2012; Read et al. 2013; Westbroek et al. 1993). While the calcifying diploid life-cycle stage of this species has been intensively studied in field and laboratory experiments, the non-calcifying haploid stage has only recently gained attention due to its important ecological role. In blooms of diploid *E.* *huxleyi*, which are usually terminated by viruses, the haploid life-cycle stage functions as a virus-resistant backup population (Frada et al. 2012). Furthermore, the presence and absence of calcification in the differing life-cycle stages of *E. huxleyi* make them ideal candidates to investigate the cellular mechanisms of calcification and their interaction with photosynthesis under increasing oceanic CO_2_ concentrations (Mackinder et al. 2010; Rokitta and Rost 2012).

Increasing *p*CO_2_ in oceanic surface water directly affects carbonate chemistry by elevating the concentration of dissolved inorganic carbon (DIC) and shifting the carbon speciation toward higher CO_2_ and H^+^ concentrations, a phenomenon often referred to as ocean acidification (OA; Caldeira and Wickett 2003; Wolf-Gladrow et al. 1999). Compared to preindustrial values, pH is expected to drop by 0.4–0.5 units until the end of this century. In several studies testing the effects of OA on *E. huxleyi*, diploid strains were found to exhibit strong, yet opposing responses in terms of biomass and calcite production. While biomass production was either unaffected or stimulated by increased *p*CO_2_, calcification typically decreased and malformations of coccoliths increased (e.g., Hoppe et al. 2011; Langer et al. 2009; Riebesell et al. 2000). Bach et al. (2011) suggested that biomass production is stimulated by increasing CO_2_ concentration at sub-saturating conditions, whereas calcification is specifically responsive to the associated decrease in pH. Such differential CO_2_ and pH effects on biomass and calcite production are supported by the observation that OA distorts ion homeostasis and shifts the metabolism from oxidative to reductive pathways (Rokitta et al. 2012; Taylor et al. 2011). In a number of studies, the sensitivity of *E. huxleyi* toward OA has been attributed to its mode of inorganic carbon (C_i_) acquisition, which is intrinsically responsive to changes in carbonate chemistry. Thus, for understanding the differential responses to OA, one needs to look at this crucial process of C_i_ assimilation.

Like most phytoplankton, *E. huxleyi* operates a CO_2_ concentrating mechanism (CCM), which utilizes CO_2_ and/or HCO_3_^−^ uptake systems to accumulate CO_2_ in the vicinity of RubisCO, and employs the enzyme carbonic anhydrase (CA) to accelerate the inter-conversion between these C_i_ species (see Reinfelder 2011 for review). For a long time, the CCM in *E.* *huxleyi* was assumed to rely on the CO_2_ delivery by calcification (Anning et al. 1996; Sikes et al. 1980). More recently, however, studies have demonstrated that C_i_ fluxes for photosynthesis and calcification are independent (Herfort et al. 2004; Rost et al. 2002; Trimborn et al. 2007), and that these two processes may even compete for C_i_ substrates (Rokitta and Rost 2012). Most studies performed on the CCM of *E. huxleyi* to date yielded moderately high substrate affinities for C_i_, which decreased slightly under OA scenarios (e.g., Rokitta and Rost 2012; Rost et al. 2003, Stojkovic et al. 2013). Moreover, low activity for extracellular CA and high contribution of HCO_3_^−^ uptake for photosynthesis have been reported (e.g., Herfort et al. 2002; Rokitta and Rost 2012; Stojkovic et al. 2013; Trimborn et al. 2007). This high apparent HCO_3_^−^ usage is puzzling, however, as it suggests biomass production to be rather insensitive to OA-related changes in CO_2_ supply, which is in contrast to what studies usually have observed.

Most physiological methods characterizing the CCM and its functional elements are performed under standardized assay conditions, including a fixed pH value, and thus differing from treatment conditions. The pH and the concominant C_i_ speciation can, however, influence the cell’s physiology, in particular its C_i_ acquisition. When identifying the cause-effect relationship in OA responses, it is difficult to separate the effects of changes in C_i_ speciation from concomitant changes in H^+^ concentrations. Changes in external pH have been shown to directly drive changes in cytosolic pH in *E. huxleyi*, which, in turn, affected H^+^ gradients and membrane potentials (Suffrian et al. 2011; Taylor et al. 2011). This effect could indirectly impact secondary active transporters, e.g., the Cl^−^/HCO_3_^−^ antiporter (Herfort et al. 2002; Rokitta et al. 2011). Moreover, the protonation of amino acid side chains can affect activity, specificity, and kinetics of enzymes and transporters involved in cellular processes (Badger 2003; Raven 2006). Hence, aside from altered concentrations of C_i_ species, pH itself could directly impact the mode of CCM (Raven 1990). These possible effects of the assay pH on C_i_ acquisition should be accounted for when performing experiments to characterize the CCM.

One common approach to determine the C_i_ source for photosynthesis is the application of the ^14^C disequilibrium method (Espie and Colman 1986), which has proven suitable for the study of marine phytoplankton in laboratory cultures (e.g., Elzenga et al. 2000; Rost et al. 2006a) and in natural field assemblages (e.g., Cassar et al. 2004; Martin and Tortell 2006; Tortell and Morel 2002; Tortell et al. 2008). The method makes use of the relatively slow chemical conversion between the CO_2_ and HCO_3_^−^ in the absence of CA (Johnson 1982), allowing for a differential labeling of these C_i_ species with ^14^C. This method is typically performed at pH of 8.5 ("assay pH"), deviating strongly from most natural in situ values and even more from the pH values applied in OA-experiments ("acclimation pH"). In this study, we aimed to disentangle the short-term effect of assay pH from the long-term effect of acclimation history on the photosynthetic C_i_ source of *E. huxleyi*. To this end, we grew haploid and diploid life-cycle stages at present-day (380 μatm) and elevated *p*CO_2_ (950 μatm), and measured the responses in growth, elemental composition, and production rates. These low and high *p*CO_2_-acclimated cells were then tested for their preferred C_i_ source by applying the ^14^C disequilibrium method, with assay conditions set to a range of ecologically relevant pH values (pH 7.9–8.7). The reliability of this new approach was tested by performing sensitivity studies.

## Methods

### *p*CO_2_ acclimations

Haploid and diploid cells of *E. huxleyi* (strains RCC 1217 and RCC 1216, obtained from the Roscoff culture collection) were grown at 15 °C as dilute batch incubations. North Sea seawater medium (salinity 32.4) was sterile-filtered (0.2 μm) and enriched with vitamins and trace metals according to F/2 (Guillard and Ryther 1962), as well as phosphate and nitrate (100 and 6.25 μmol L^−1^). Cells were exposed to a light:dark cycle (16:8 h) and saturating light (300 μmol photons m^−2^ s^−1^) provided by daylight lamps (FQ 54W/965HO, OSRAM, Munich, Germany). Light intensity was monitored with the LI-6252 datalogger (LI-COR, Lincoln, NE, USA) using a 4π-sensor (US-SQS/L, Walz, Effeltrich, Germany). Culturing was carried out in sterilized 2.4 L borosilicate bottles (Duran Group, Mainz, Germany) on roller tables to avoid sedimentation.

Prior to experiments, cells were acclimated to the respective *p*CO_2_ and light conditions for at least 7 days (i.e., more than 10 generations). Prior to initiating cultures, medium was pre-aerated for at least 36 h with humidified, 0.2 μm-filtered air comprising *p*CO_2_ values of 380 or 950 μatm (equivalent to 38.5 and 96.3 Pa, or ~15 and ~35 μmol kg^−1^, respectively). Gas mixtures were created by a gas flow controller (CGM 2000 MCZ Umwelttechnik, Bad Nauheim, Germany) using pure CO_2_ (Air Liquide Deutschland, Düsseldorf, Germany) and CO_2_-free air (CO2RP280, Dominick Hunter, Willich, Germany). Sampling and measurements were done 4–8 h after the beginning of the light period (i.e., at midday) in exponential growth at densities of 40,000–60,000 cells mL^−1^. Cultures showing a pH drift of > 0.05 were excluded from further analyses.

The carbonate system (Table 1) during the acclimations was assessed based on measurements of pH and total alkalinity (TA). The pH_NBS_ of the cultures was measured potentiometrically and corrected for temperature (pH-meter 3110; WTW, Weilheim, Germany). The electrode (A157, Schott Instruments, Mainz, Germany) was three-point calibrated with NBS certified standard buffers and the measurement uncertainty was 0.03 pH units. TA was determined by potentiometric titration (Dickson 1981; TitroLine alpha plus, Schott Instruments). Measurements were accuracy-corrected with certified reference materials (CRMs) supplied by A. Dickson (Scripps Institution of Oceanography, USA). Calculation of the carbonate system was performed using CO2sys (Pierrot et al. 2006). Input parameters were pH_NBS_ and TA, as well as temperature (15 °C), salinity (32.4), and pressure (1 dbar, according to 1 m depth; Hoppe et al. 2012). For all calculations, phosphate and silicate concentrations were assumed to be 7 and 17 μmol kg^−1^, respectively, based on assessments of the media. Equilibrium constants for carbonic acid, K_1_ and K_2_ given by Mehrbach et al. (1973) and refit by Dickson and Millero (1987) were used. For the dissociation of sulfuric acid, the constants reported by Dickson (1990) were employed.

**Table 1:** Carbonate chemistry of the *p*CO_2_ acclimations at the time of harvesting and in cell-free media (reference); Attained *p*CO_2_, DIC, HCO_3_^−^, CO_3_^2−^, and Ω_calcite_ are calculated based on measured pH_NBS_ and TA using CO2sys (Pierrot et al. 2006)

Strain, ploidy	Treatment *p*CO_2_ (μatm)	Attained *p*CO_2_ (μatm)	pH_NBS_	TA (μmol kg^−1^)	DIC (μmol kg^−1^)	CO_2_ (μmol kg^−1^)	HCO_3_^−^ (μmol kg^−1^)	CO_3_^2−^ (μmol kg^−1^)	Ω_calcite_
RCC 1216, 2N	Low, 380	353 ± 8	8.19 ± 0.02	2,259 ± 19	2,023 ± 15	13 ± 0	1,857 ± 13	161 ± 3	3.9 ± 0.1
	High, 950	847 ± 55	7.86 ± 0.04	2,278 ± 20	2,156 ± 2	32 ± 2	2,060 ± 28	84 ± 4	2.0 ± 0.1
RCC 1217, 1N	Low, 380	345 ± 4	8.23 ± 0.00	2,317 ± 12	2,068 ± 10	13 ± 0	1,885 ± 10	170 ± 1	4.1 ± 0.0
	High, 950	837 ± 25	7.89 ± 0.01	2,317 ± 3	2,210 ± 5	32 ± 1	2,092 ± 5	86 ± 3	2.1 ± 0.1
Cell-free medium	Low, 380	405 ± 3	8.17 ± 0.00	2,304 ± 5	2,092 ± 5	15 ± 0	1,926 ± 5	151 ± 1	3.7 ± 0.0
	High, 950	997 ± 17	7.82 ± 0.01	2,305 ± 7	2,214 ± 12	38 ± 1	2,128 ± 11	75 ± 1	1.8 ± 0.0

Results are reported for 15 °C (*n* ≥ 3; ± SD)

Cell growth was assessed by daily cell counting with a Multisizer III hemocytometer (Beckman-Coulter, Fullerton, CA, USA) and the specific growth rates (*μ*) were calculated from daily increments (cf., Rokitta and Rost 2012). For the determination of total particulate carbon (TPC), POC and particulate organic nitrogen (PON), cell suspensions were vacuum-filtered (-200 mbar relative to atmosphere) onto pre-combusted (12 h, 500 °C) GF/F filters (1.2 μm; Whatman, Maidstone, UK), which were dried at 65 °C and analyzed with a EuroVector CHNS-O elemental analyzer (EuroEA, Milano, Italy). Before quantification of POC, filters were HCl-soaked (200 μL, 0.2 M) and dried to remove calcite. PIC was assessed as the difference between TPC and POC. By multiplying the POC and PIC cell quotas with *μ*, the respective production rates were derived (cf., Rokitta and Rost 2012). For Chl *a* measurements, cells were filtered onto cellulose nitrate filters (0.45 μm; Sartorius, Göttingen, Germany) and instantly frozen in liquid nitrogen. Chl *a* was extracted in 90 % acetone (v/v, Sigma, Munich, Germany) and determined fluorometrically (TD-700 fluorometer, Turner Designs, Sunnyvale, USA) following the protocol by Holm-Hansen and Riemann (1978). The calibration of the fluorometer was carried out with a commercially available Chl *a* standard (*Anacystis nidulans*, Sigma, Steinheim, Germany).

### ^14^C disequilibrium method

**Fig. 1:**
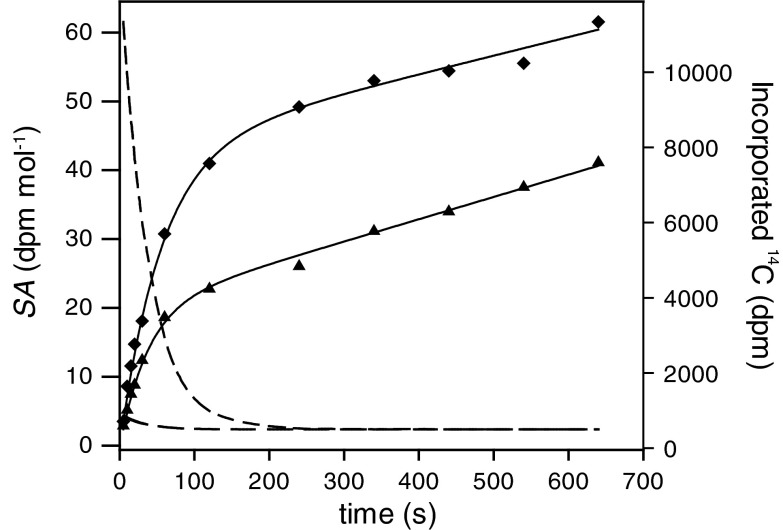
Time-course of specific activities of CO_2_ and HCO_3_^−^ (medium and long dashed lines, respectively, here calculated for assay pH 8.5) in the isotopic disequilibrium method and examples for the ^14^C incorporation of the diploid life-cycle stage for predominant CO_2_ usage ($f_{{{\text{CO}}_{ 2} }} = 1.00$, *squares*) and considerable HCO_3_^−^ usage ($f_{{{\text{CO}}_{ 2} }} = 0.60$, *triangles*)

The C_i_ source for photosynthesis was determined by applying the ^14^C disequilibrium method (Elzenga et al. 2000; Espie and Colman 1986; Tortell and Morel 2002). In this method, a transient isotopic disequilibrium is induced by adding a small volume of a ^14^C_i_ "spike" solution with a relatively low pH (typically 7.0) into larger volume of buffered cell suspension with a relatively high pH (typically 8.5). The cell suspension contains dextran-bound sulfonamide (DBS) to eliminate possible external CA activity. Due to the pH-dependent speciation of DIC, the relative CO_2_ concentration of the spike is high (~19 % of DIC at pH 7.0), compared to the cell suspension (~0.3 % of DIC at pH 8.5). When adding the spike to the cell suspension, the majority of the CO_2_ added with the spike converts into HCO_3_^−^ until equilibrium is achieved (Johnson 1982; Millero and Roy 1997). Consequently, the specific activity of CO_2_ (${\text{SA}}_{{{\text{CO}}_{2} }}$, dpm (mol CO_2_)^−1^) is initially high and exponentially decays over time (Fig. 1). The slope of the ^14^C incorporation curve of a "CO_2_ user" is, therefore, initially much steeper than during final linear ^14^C uptake, when isotopic equilibrium is achieved. In contrast, the slope of ^14^C incorporation for "HCO_3_^−^ users" changes only marginally over time because ${\text{SA}}_{{{\text{HCO}}_{3}^{ - } }}$ stays more or less constant during the assay.

Quantification of the relative proportion of CO_2_ or HCO_3_^−^ usage was done by fitting data with the integral function of the ^14^C fixation rate (Elzenga et al. 2000; Espie and Colman 1986; Martin and Tortell 2006). The function includes terms representing the instantaneous fixation rate of DI^14^C, the fractional contribution of CO_2_ $\left( {f_{{{\text{CO}}_{2} }} } \right)$ or HCO_3_^−^ usage $\left( {1 - f_{{{\text{CO}}_{2} }} } \right)$ to the overall C_i_ fixation and the specific activity (SA, dpm mol^−1^) of these substrates at any given time (Eq. 1; Espie and Colman 1986; Elzenga et al. 2000; Tortell and Morel 2002). Strictly speaking, as HCO_3_^−^ and CO_3_^2−^ cannot be differentially labeled, $1 - f_{{{\text{CO}}_{2} }}$ also comprises the potential fraction of CO_3_^2−^ used.

1 $$\begin{aligned} {\text{dpm}} = & V_{\text{DI14C}} \left( {f_{{{\text{CO}}_{2} }} } \right){{\left( {\alpha_{1} t + \left( {\Delta {\text{SA}}_{{{\text{CO}}_{2} }} / {\text{SA}}_{\text{DIC}} } \right)\left( {1 - e^{{ - \alpha_{1} t}} } \right)} \right)} \mathord{\left/ {\vphantom {{\left( {\alpha_{1} t + \left( {\Delta {\text{SA}}_{{{\text{CO}}_{2} }} / {\text{SA}}_{\text{DIC}} } \right)\left( {1 - e^{{ - \alpha_{1} t}} } \right)} \right)} {\alpha_{1} }}} \right. \kern-0pt} {\alpha_{1} }}+ V_{\text{DI14C}} \left( {1 - f_{{{\text{CO}}_{2} }} } \right){{\left( {\alpha_{2} t + \left( {\Delta {\text{SA}}_{{{\text{HCO}}_{3} }} / {\text{SA}}_{\text{DIC}} } \right)\left( {1 - e^{{ - \alpha_{2} t}} } \right)} \right)} \mathord{\left/ {\vphantom {{\left( {\alpha_{2} t + \left( {\Delta {\text{SA}}_{{{\text{HCO}}_{3} }} / {\text{SA}}_{\text{DIC}} } \right)\left( {1 - e^{{ - \alpha_{2} t}} } \right)} \right)} {\alpha_{2} }}} \right. \kern-0pt} {\alpha_{2} }} \\ \end{aligned}$$

In this equation, *V*_DI14C_ is the total rate of ^14^C uptake; $f_{{{\text{CO}}_{ 2} }}$ is the fraction of uptake attributable to CO_2_; *α*_1_ and *α*_2_ are the temperature-, salinity-, and pH-dependent first-order rate constants for CO_2_ and HCO_3_^−^ hydration and dehydration, respectively; *t* is the time (s); $\Delta {\text{SA}}_{{{\text{CO}}_{2} }}$ and $\Delta {\text{SA}}_{{{\text{HCO}}_{3} }}$ are the differences between the initial and equilibrium values of the specific activities of CO_2_ and HCO_3_^−^, respectively; and SA_DIC_ is the specific activity of DIC. During steady-state photosynthesis, V_DI14C_ and $f_{{{\text{CO}}_{ 2} }}$ are assumed to be constant so that changes in the instantaneous ^14^C uptake rate reflect only changes in the specific activity of CO_2_ and HCO_3_^−^.

In the present study, the ^14^C disequilibrium method was modified to enable measurements over a range of ecologically relevant pH values (7.90–8.70). In order to maintain a suitably large initial isotopic disequilibrium $\left( {\Delta {\text{SA}}_{{{\text{CO}}_{2} }} / {\text{SA}}_{\text{DIC}} } \right)$, the pH of the ^14^C spike solutions needs to be adjusted in conjunction with the pH of the assay buffer. We, thus, used either MES or HEPES buffers to set the pH of spike solutions over the range of 5.75–7.30 (see Table 2 for exact pH values of assay and spike buffers). For the assays, 10–30 × 10^6^ cells were concentrated via gentle filtration over a polycarbonate filter (2 μm; Millipore, Billerica, MA, USA) to a final volume of 15 mL. During this filtration procedure, cells were kept in suspension, while the medium was gradually exchanged with buffered assay medium of the appropriate pH value. Assay media and spike buffers were prepared at least 1 day prior to the assay and stored in closed containers to avoid CO_2_ exchange and pH drift. The pH value and temperatures of all buffers were measured immediately prior to assay runs. DIC concentration of the assay buffers was determined colorimetrically according to Stoll et al. (2001) using a TRAACS CS800 autoanalyzer (Seal Analytical, Norderstedt, Germany), and measurements were accuracy-corrected with CRMs supplied by A. Dickson (Scripps Institution of Oceanography, USA).

**Table 2:** Chemical characteristics of ^14^C disequilibrium assay media and spike buffers, and the associated parameter values for model fits (Eq. 1)

Assay medium			Spike solution			Conditions for RCC 1216, 2N						Conditions for RCC 1217, 1N					
pH	Buffer chemical	CO_2_ (%)	pH	Buffer chemical	CO_2_ (%)	DIC (μM)	CO_2_ (μM)	*α*_1_	*α*_2_	$\frac{{\Delta {\text{SA}}_{{{\text{CO}}_{ 2} }} }}{{{\text{SA}}_{\text{DIC}} }}$	$\frac{{\Delta {\text{SA}}_{{{\text{HCO}}_{ 3}^{ - } }} }}{{{\text{SA}}_{\text{DIC}} }}$	DIC (μM)	CO_2_ (μM)	*α*_1_	*α*_2_	$\frac{{\Delta {\text{SA}}_{{{\text{CO}}_{ 2} }} }}{{{\text{SA}}_{\text{DIC}} }}$	$\frac{{\Delta {\text{SA}}_{{{\text{HCO}}_{ 3}^{ - } }} }}{{{\text{SA}}_{\text{DIC}} }}$
7.90	BICINE	1.1	5.75	MES	80.4	2,210	23.4	0.0186	0.0197	29.09	−0.786	2,490	26.7	0.0176	0.0186	28.44	−0.786
8.10	BICINE	0.7	6.35	MES	50.7	2,250	14.6	0.0205	0.0225	30.08	−0.451	2,680	17.6	0.0194	0.0212	30.09	−0.454
8.30	BICINE	0.4	6.70	MES	31.5	2,290	8.9	0.0236	0.0272	30.46	−0.204	2,590	10.3	0.0223	0.0256	29.83	−0.206
8.50	BICINE	0.2	7.00	HEPES	18.7	2,380	5.4	0.0285	0.0355	31.37	−0.012	2,310	5.4	0.0270	0.0334	27.87	0.008
8.70	BICINE	0.1	7.30	HEPES	10.3	2,150	2.8	0.0364	0.0504	29.16	−0.237	–	–	–	–	–	–

Assays with the diploid cells (2N) were conducted at an assay temperature of 15.5 °C, a spike temperature of 23 °C, an added radioactivity of 315 kBq and a salinity of 32.4. Assays with the haploid cells (1N) were conducted at an assay temperature of 15.0 °C, a spike temperature of 23 °C, a spike radioactivity of 370 kBq and a salinity of 32.4

To initiate the assays, a volume of 4 mL buffered concentrated cell suspension was transferred into a temperature-controlled, illuminated glass cuvette (15 °C; 300 μmol photons m^−2^ s^−1^) to which 50 μM DBS was added (Ramidus, Lund, Sweden). Cells were continuously stirred in the light for at least 5 min prior to spike addition to reach steady-state photosynthesis. Spike solutions were prepared by adding NaH^14^CO_3_ solution (1.88 GBq (mmol DIC)^−1^; GE Healthcare, Amersham, UK) into a final volume of 200 μL of pH-buffered MilliQ water (various buffers at 20 mM; Table 2), yielding activities of ~370 kBq (10 μCi). Following the spike addition, 200 μL subsamples of the cell suspension were transferred into 2 mL HCl (6 M) at time points between 5 s and 12 min. Addition of these aliquots to the strong acid caused instant cell death and converted all DIC and PIC to CO_2_. DI^14^C background was degassed in a custom-built desiccator for several days until samples were dry. Deionized water (1 mL) was then added to re-suspend samples prior to addition of 10 mL of scintillation cocktail (Ultima Gold AB, GMI, Ramsey, MN, USA), and the sample was vortexed thoroughly.

Acid-stable (i.e., organic) ^14^C activity in samples was counted with a Packard Tri-Carb Liquid Scintillation Counter (GMI). Blank samples, consisting of cell-free medium, were treated alongside the other samples. In the few cases where no blanks were available, time zero values were approximated by extrapolating the y-axis intercept from linear fitting of the first three data points of the ^14^C incorporation curves. Total radioactivity of the NaH^14^CO_3_ stock solution was regularly quantified and compared to expected values to estimate loss of radioactivity or changes in counting efficiency. In all spike solutions, measured radioactivity ranged between 80 and 100 % of the theoretical values, and the actual radioactivity levels were used in the calculation of the specific activities. Blank-corrected data were fitted (Eq. 1), using a least-squares-fitting procedure. Applied fit parameters are given in Table 2. Furthermore, a detailed Excel spread sheet for calculating the fit parameters in dependence of the applied conditions (e.g., pH, temperature and DIC concentrations) is provided as Supplementary Material. Please note that in the calculation of initial and final specific activities, we accounted not only for changes in concentrations of ^14^C_i_ species but also for changes in concentrations of DI^12^C, ^12^CO_2_, and H^12^CO_3_^−^ upon spike addition. If these changes are neglected, $\Delta {\text{SA}}_{{{\text{CO}}_{2} }} / {\text{SA}}_{\text{DIC}}$ will be significantly overestimated, leading to an underestimation of $f_{{{\text{CO}}_{ 2} }}$ (Eq. 1, Table 2, Supplementary material).

We used a numerical sensitivity study to examine how offsets in parameters such as pH, DIC concentrations, radioactivity, temperature, or blank values influence the derived estimates of $f_{{{\text{CO}}_{ 2} }}$. First, theoretical ^14^C incorporation curves for "HCO_3_^−^ users" $\left( {f_{{{\text{CO}}_{ 2} }} = 0.25} \right)$ and "CO_2_ users" $\left( {f_{{{\text{CO}}_{ 2} }} = 0.80} \right)$ were generated for two assay pH values (7.90 and 8.50) and used as a reference, assuming fixed values of DIC concentrations of 2,300 μmol kg^−1^, assay temperature of 15 °C, spike solution temperature of 23 °C and spike radioactivity of 370 kBq. In a second step, model fits were obtained using slight offsets in these parameters (e.g., pH 7.95 and 7.85 instead of 7.90) to obtain the effect of parameter variability on $f_{{{\text{CO}}_{ 2} }}$ estimates. Sensitivity toward over- and underestimation of pH, temperature, DIC concentration, and radioactivity was tested. We further assessed the effects of blank values (±100 dpm) on $f_{{{\text{CO}}_{ 2} }}$ estimates as a function of different final ^14^C incorporation rates.

### Statistics

All experiments were performed using at least biological triplicates (i.e., three independent, but equally treated cultures). When data were normally distributed (Shapiro-Wilk test) and showed equal variance (Equal-Variance Test), significance in difference between *p*CO_2_ treatments was tested by performing student′s t-tests. When samples were not normally distributed or did not show equal variance, a rank sum test was performed instead. Null hypotheses were rejected when *p* ≤ 0.05, unless otherwise indicated.

## Results

In diploid cells of *E. huxleyi*, the specific growth rate *μ* and PIC quotas did not change significantly in response to elevated *p*CO_2_ (Table 3). While there was a small decrease in PIC production rates (−11 %), POC quotas and production rates increased strongly under elevated *p*CO_2_ (+77 and +55 %, respectively). In conjunction with these changes, the quotas and production rates of TPC also increased (+28 and +23 %, respectively). The PIC:POC ratios of diploid cells decreased from 1.4 to 0.7 under elevated *p*CO_2_, while the POC:PON ratios increased from 6.3 to 8.8. Chl *a* quotas were largely unaffected by the *p*CO_2_ treatments, although Chl *a*:POC ratios decreased significantly from 0.022 to 0.012 pg pg^−1^ under elevated *p*CO_2_, owing to the change in POC quotas. In haploid cells, neither *μ*, elemental quotas or the respective production rates showed any significant response to elevated *p*CO_2_ (Table 3). Similarly, Chl *a* quotas, Chl *a*:POC, and POC:PON ratios were all unaffected by the experimental CO_2_ manipulations in the haploid strain.

**Table 3:** Growth rates, elemental quotas and production rates, elemental ratios, as well as pigment composition of haploid (1N) and diploid (2N) cells of *E. huxleyi*, cultured at low (380 μatm) and elevated *p*CO_2_ (950 μatm): *μ* (day^−1^), POC quota (pg cell^−1^), POC production (pg cell^−1^ day^−1^), PIC quota (pg cell^−1^), PIC production (pg cell^−1^ day^−1^), TPC quota (pg cell^−1^), TPC production (pg cell^−1^ day^−1^), PON quota (pg cell^−1^), PON production (pg cell^−1^ day^−1^), PIC:POC ratio (mol:mol), POC:PON ratio (mol:mol), Chl *a* quotas (pg cell^−1^), and Chl *a*:POC ratios (pg:pg)

Parameter	1N low *p*CO_2_	1 N high *p*CO_2_	*p*	2N low *p*CO_2_	2N high *p*CO_2_	*p*
*μ*	1.12 ± 0.04	1.08 ± 0.06	†	1.08 ± 0.05	1.04 ± 0.04	†
POC quota	10.76 ± 0.23	11.08 ± 1.19	†	8.35 ± 0.84	14.78 ± 1.91	**
POC production	12.09 ± 0.25	12.81 ± 0.44	†	9.02 ± 0.91	13.97 ± 0.63	*
PIC quota	0.48 ± 0.43	−0.18 ± 0.21	†	11.78 ± 0.78	10.90 ± 0.60	†
PIC production	–	–	†	12.71 ± 0.29	11.35 ± 0.90	**
TPC quota	11.23 ± 0.66	12.01 ± 1.27	†	20.13 ± 1.34	25.68 ± 2.00	*
TPC production	12.63 ± 0.70	12.51 ± 0.52	†	21.73 ± 1.05	26.77 ± 3.10	≤ 0.06
PON quota	1.39 ± 0.06	1.45 ± 0.09	†	1.54 ± 0.12	1.95 ± 0.22	*
PON production	1.56 ± 0.06	1.56 ± 0.08	†	1.66 ± 0.10	2.03 ± 0.30	†
PIC:POC	–	–	†	1.42 ± 0.14	0.75 ± 0.11	**
POC:PON	9.03 ± 0.19	8.90 ± 0.69	†	6.31 ± 0.30	8.83 ± 0.17	***
Chl *a* quota	0.10 ± 0.01	0.12 ± 0.01	†	0.18 ± 0.01	0.17 ± 0.01	†
Chl *a :*POC	0.009 ± 0.001	0.012 ± 0.001	†	0.022 ± 0.001	0.012 ± 0.001	***

For the haploid cells, PIC production and PIC:POC ratios were not calculated. Stars indicate statistical significance levels in differences between low and high *p*CO_2_ treatments with * *p* ≤ 0.05, ** *p* ≤ 0.01 and *** *p* ≤ 0.001. No significant difference (*p* > 0.05) is indicated by †

**Fig. 2:**
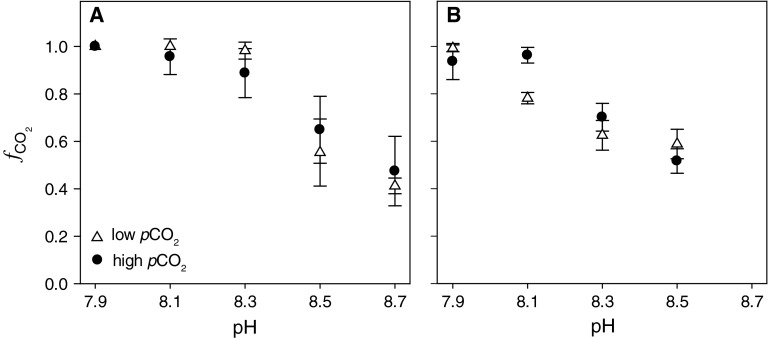
Fraction of CO_2_ usage $\left( {f_{{{\text{CO}}_{ 2} }} } \right)$ as a function of the assay pH in **A** the diploid *E. huxleyi* RCC 1216 and **B** the haploid RCC 1217 being acclimated to low *p*CO_2_ (380 μatm, *white triangles*) and high *p*CO_2_ (950 μatm, *black circles*)

Under both *p*CO_2_ acclimations, diploid cells were shown to be predominant "CO_2_ users" under low assay pH ($f_{{{\text{CO}}_{ 2} }}$ ~ 1.0 at pH 7.9; Fig. 2a). With increasing assay pH, however, we observed a significant increase in relative HCO_3_^−^ utilization. HCO_3_^−^ uptake was induced at assay pH ≥ 8.3 (equivalent to CO_2_ concentrations ≤ 9 μmol L^−1^), reaching considerable contribution at high assay pH ($f_{{{\text{CO}}_{ 2} }}$ ~ 0.44 at pH 8.7). In contrast to the strong effect of the assay pH, the tested *p*CO_2_ acclimations had no effect on the pH-dependent C_i_ uptake behavior (Fig. 2a). In other words, both low and high *p*CO_2_-acclimated cells showed the same short-term response of $f_{{{\text{CO}}_{ 2} }}$ to assay pH. Like the diploid stage, haploid cells progressively changed from high CO_2_ usage at low assay pH ($f_{{{\text{CO}}_{ 2} }}$ ~ 0.96 at pH 7.9) to substantial HCO_3_^−^ contributions when assays were conducted in high pH assay buffers ($f_{{{\text{CO}}_{ 2} }}$ ~ 0.55 at pH 8.5; Fig. 2b). HCO_3_^−^ uptake became relevant at pH ≥ 8.1 (equivalent to CO_2_ concentrations ≤ 14 μmol L^−1^), particularly in low *p*CO_2_-acclimated cells. Except for haploid cells measured at pH 8.1, no significant differences in $f_{{{\text{CO}}_{ 2} }}$ were observed between the low and high *p*CO_2_ acclimations (Fig. 2b).

**Fig. 3:**
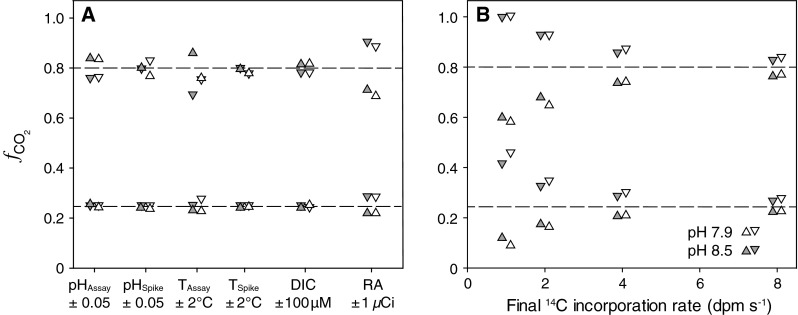
Sensitivity in $f_{{{\text{CO}}_{ 2} }}$ estimates for "CO_2_ users" ($f_{{{\text{CO}}_{ 2} }} = 0.80$) and "HCO_3_^−^ users" ($f_{{{\text{CO}}_{ 2} }} = 0.25$) at low pH (7.9, *in* *gray*) and high pH (8.5, *in* *white*) **A** toward negative (*inverted filled triangle*) and positive (*filled triangle*) offsets in the pH, temperature, and DIC concentration of the assay buffer (pH_Assay_, *T*_Assay_, and [DIC]), as well as toward offsets pH, temperature, and radioactivity of the spike (pH_Spike_, *T*_Spike_, and RA), and **B** toward negative (*inverted filled triangle*) and positive (*filled triangle*) offsets in blank measurements (±100 dpm) in dependence of the final ^14^C incorporation rates. Sensitivity was assessed based on theoretical curves with constraints of a [DIC]_Assay_ = 2,300 μM, *T*_Assay_ = 15 °C, *T*_Spike_ = 23 °C, and RA_Spike_ = 37 kBq. *Dashed lines* indicate $f_{{{\text{CO}}_{ 2} }}$ values as expected for optimal experimental conditions

The sensitivity analysis showed that an offset in the input pH of the buffered assay cell suspension (± 0.05 pH units) led to deviations in $f_{{{\text{CO}}_{ 2} }}$ of ≤ 0.09 (i.e., 9 percentage points) in "CO_2_ users" and ≤ 0.02 in "HCO_3_^−^ users" (Fig. 3a). An offset in the input temperature of the assay buffer (± 2 °C) led to a deviation in $f_{{{\text{CO}}_{ 2} }}$ of ≤ 0.09 in "CO_2_ users" and ≤ 0.03 in "HCO_3_^−^ users" (Fig. 3a). An offset in the input pH of the spike (± 0.05 pH units) changed the $f_{{{\text{CO}}_{ 2} }}$ estimates by ≤ 0.08 in "CO_2_ users" and ≤ 0.03 in "HCO_3_^−^ users" (Fig. 3a). Applying an offset in the input temperature of the spike (± 2 °C) caused a deviation in $f_{{{\text{CO}}_{ 2} }}$ by ≤ 0.06 in "CO_2_ users" and had practically no effect on $f_{{{\text{CO}}_{ 2} }}$ in "HCO_3_^−^ users" (≤ 0.01; Fig. 3a). An offset in the input DIC concentration of the buffer (± 100 μmol kg^−1^) affected $f_{{{\text{CO}}_{ 2} }}$ by ≤ 0.08 in "CO_2_ users" and ≤ 0.03 in "HCO_3_^−^ users". Regarding the radioactivity of the spike (± 37 kBq), deviations in $f_{{{\text{CO}}_{ 2} }}$ were ≤ 0.12 in "CO_2_ users" and ≤ 0.04 in "HCO_3_^−^ users." Irrespective of CO_2_ or HCO_3_^−^ usage, offsets in blank estimations (± 100 dpm) led to deviating $f_{{{\text{CO}}_{ 2} }}$ by ≤ 0.27, but only when equilibrium ^14^C fixation rates were ≤ 1 dpm s^−1^ (Fig. 3b). When steady-state ^14^C incorporation rates were ≥ 2 dpm s^−1^ (i.e., average rate in diploid cells) and ≥ 4 dpm s^−1^ (i.e., average rate in haploid cells), the deviations in $f_{{{\text{CO}}_{ 2} }}$ due to offsets in the blanks were ≤ 0.17 and ≤ 0.11, respectively.

## Discussion

### Acclimation responses

This study corroborates previous findings on the general sensitivity of the diploid life-cycle stage of *E.* *huxleyi* toward OA (e.g., Feng et al. 2008; Langer et al. 2009; Riebesell et al. 2000). While growth rate was unaffected, OA reduced PIC production and stimulated POC production (Table 3). Consequently, the PIC:POC ratio was strongly decreased under OA, indicating a redirection of C_i_ fluxes between these two processes. Transcriptomics have previously attributed this redirection to an inhibition of calcification in response to impaired signal-transduction and ion-transport, as well as to stimulation in the production of glycoconjugates and lipids (Rokitta et al. 2012). In our study, also the TPC production increased significantly under OA (Table 3), indicating that not only C_i_ is allocated differently, but also the overall C_i_ uptake increases with the increasing *p*CO_2_. Our data further suggest that less energy is required for the C_i_ acquisition under OA as more POC and TPC could be produced even though the Chl *a* quota remained unaffected by the *p*CO_2_ treatment (Table 3). Improved energy-use efficiencies under OA have previously been proposed for the diploid life-cycle stage of *E.* *huxleyi* (Rokitta and Rost 2012).

In strong contrast to the diploid strain, the haploid life-cycle stage of *E. huxleyi* was insensitive toward OA with respect to growth rate and elemental composition (Table 3). The ability of the haploid cells to maintain homeostasis under OA has also been observed by Rokitta and Rost (2012). Even though the haploid cells appeared non-responsive toward OA on the phenomenological level (i.e., growth, elemental composition), transcriptomics have revealed significant changes at the subcellular level, such as an upregulation of catabolic pathways under OA (Rokitta et al. 2012). Based on the comparison of the life-cycle stages, Rokitta and co-workers concluded that the OA sensitivity in diploid cells originates from calcification, differences in C_i_ acquisition or both.

A number of studies have shown that *E. huxleyi* has moderately high C_i_ affinities and uses HCO_3_^−^ as the primary C_i_ source (e.g., Herfort et al. 2002; Rokitta and Rost 2012; Rost et al. 2006b; Stojkovic et al. 2013), irrespective of the degree of calcification (Trimborn et al. 2007; Rokitta and Rost 2012). These characteristics would suggest *E. huxleyi* to be rather insensitive toward OA and the associated rise in CO_2_ concentration, contrary to most results obtained for the diplont. As discussed below, this apparent discrepancy could originate from differences in conditions applied during short-term physiological measurements and those conditions cells experience in the long-term acclimation.

### Modes of C_i_ acquisition

Our results demonstrate that the C_i_ source of both life-cycle stages of *E. huxleyi* is significantly influenced by the pH of the assay medium and the resulting carbonate chemistry (Fig. 2). With increasing pH in assay buffers, cells progressively changed from predominant CO_2_ usage at lower pH values (≤ 8.1) to significant HCO_3_^−^ contribution at higher pH (≥ 8.3). Surprisingly, this change occurred irrespectively of the *p*CO_2_ conditions in the acclimation. To our knowledge, such a strong short-term pH-dependence in C_i_ acquisition has not been previously reported, which is most likely due to the fact that assays are typically performed under standardized pH values. Measuring physiological responses under one reference condition have the advantage that consequences of different acclimations can readily be compared in terms of altered capacities of certain processes, e.g., enzyme activities or transport rates. However, determination of the C_i_ source at one standard pH appears to impose a methodological bias, and our results, therefore, bear direct relevance to the interpretation of previous laboratory observations.

In view of the short-term pH effect on C_i_ acquisition, the contribution of HCO_3_^−^ as a photosynthetic C_i_ source in *E.* *huxleyi* may have possibly been overestimated in previous studies. This overestimation is likely to be the most significant in those studies when ^14^C disequilibrium assays were conducted at pH 8.5 (e.g., Rokitta and Rost 2012; Rost et al. 2007). By looking at the C_i_ source determined at an assay pH mimicking the acclimation condition, we can now re-evaluate and in fact explain the responses of *E. huxleyi* toward elevated *p*CO_2_. When assessing $f_{{{\text{CO}}_{ 2} }}$ using assay buffers of pH 7.9 and 8.1 (equivalent to the acclimation pH of high and low *p*CO_2_ treatments), we observed predominant CO_2_ uptake under both conditions (Fig. 2). Being "CO_2_ user", cells were thus able to directly benefit from changes in the CO_2_ concentrations in our acclimations (~15 μmol kg^−1^ at 380 μatm and ~38 μmol kg^−1^ at 950 μatm). For a "HCO_3_^−^ user", however, it would be difficult to argue for a beneficial OA-effect as HCO_3_^−^ concentrations do not differ much between treatments (~1,930 μmol kg^−1^ at 380 μatm and ~2,130 μmol kg^−1^ at 950 μatm). Our results thus suggest that biomass production in diploid cells not only profits from the declined calcification at high *p*CO_2_, as suggested by Rokitta and Rost (2012) but also from the higher CO_2_ supply under OA. As CO_2_ usage is considered to be less costly than HCO_3_^−^ uptake (Raven 1990), this could also explain the higher energy-use efficiency observed for *E. huxleyi* (Rokitta and Rost 2012).

Although the haploid life-cycle stage of *E.* *huxleyi* exhibited a pH-dependent C_i_ uptake behavior that was similar to the diploid (Fig. 2), the haploid cells did not show any CO_2_-dependent stimulation in biomass production (Table 3). This could partly be related to the fact that the biomass production cannot profit from a down-scaling of calcification, simply because this process is absent in the haploid life-cycle stage. The lack of significantly stimulated biomass buildup under OA could also be attributed to the concomitant upregulation of catabolic pathways, such as higher lipid consumption, which is a specific feature of the haploid cells (Rokitta et al. 2012). After all, the similar C_i_ uptake behavior of both life-cycle stages confirms that photosynthetic HCO_3_^−^ usage is not tied to calcification (Herfort et al. 2004; Trimborn et al. 2007; Bach et al. 2013) and that the preference for CO_2_ or HCO_3_^−^ is predominantly controlled by carbonate chemistry.

Our findings clearly demonstrate that the acclimation history, in both life-cycle stages, has little or no effect on the C_i_ usage of the cells (Fig. 2). In other words, the instantaneous effect of the assay conditions dominates over acclimation effects. We cannot preclude, however, that cells acclimated to higher pH values, where CO_2_ supply becomes limiting, may increase their capacity for HCO_3_^−^ uptake and acclimations effects would then be evident. Notwithstanding the potential for some acclimation effects, the extent to which short-term pH and/or CO_2_ levels in the assay medium directly control cellular C_i_ usage is striking. This implies that even though *E. huxleyi* did not use significant amounts of HCO_3_^−^ for photosynthesis, it must constitutively express a HCO_3_^−^ transporter in all acclimations. Without the presence of a functional HCO_3_^−^ transport system we could otherwise not explain the capacity for significant HCO_3_^−^ uptake under short-term exposure to high pH (even in high *p*CO_2_-acclimated cells).

In the diploid life-cycle stage, HCO_3_^−^ transporter may be constitutively expressed to fuel calcification, as HCO_3_^−^ was identified as the main C_i_ source for this process (Paasche 1964; Rost et al. 2002; Sikes et al. 1980). If CO_2_ supply for photosynthesis becomes limiting, HCO_3_^−^ transport could then also fuel photosynthesis. In the haploid cells, which do not calcify, we nonetheless observed the same capacity for HCO_3_^−^ uptake, which suggests that HCO_3_^−^ uptake capacity represents a fundamental component of the CCM of both life-cycle stages of *E.* *huxleyi*. Whether levels of protons or CO_2_ concentrations are the main trigger for the shift between CO_2_ and HCO_3_^−^ uptake remains unclear, even though there is strong evidence that CO_2_ supply is the main driver for the responses in photosynthesis (Bach et al. 2011).

### Sensitivity analyses

In our sensitivity study, the applied offsets in pH (± 0.05 pH units), temperature (± 2 °C), DIC of the assay buffer (± 100 μM), and spike radioactivity (± 37 kBq) were larger than typical measurement errors to represent "worst-case scenarios". None of these offsets caused $f_{{{\text{CO}}_{ 2} }}$ estimates to deviate by more 0.12 in any of the pH treatments (Fig. 3a). When adequate efforts are taken to control these parameters (e.g., using reference buffers, thermostats), methodological uncertainties are thus negligible. DIC concentrations and radioactivity, however, are often not measured and in view of the potential drift over time, offsets can easily exceed typical measurement errors and lead to severe deviations in $f_{{{\text{CO}}_{ 2} }}$. For instance, ^14^CO_2_ out-gassing causes the spike solution to progressively lose radioactivity. This loss of ^14^C can easily be > 20 % over the course of weeks or months, despite the high pH values of the stock solution and small headspace in the storage vial (Gattuso et al. 2010).

The average final ^14^C fixation rates, which depend on the biomass and radioactivity used, were 2.1 ± 0.8 dpm s^−1^ in the runs with diploid and 6.6 ± 2.2 dpm s^−1^ in those with haploid cells (Fig. 3b). In these ranges, offsets in blank values (± 100 dpm) can lead to biases in the estimated $f_{{{\text{CO}}_{ 2} }}$ by up to 0.20 (Fig. 3b). This strong sensitivity highlights the need to thoroughly determine blank values, but also to work with sufficiently high biomass and/or radioactivity to maximize ^14^C incorporation rates. When working with dense cell suspensions, however, self-shading or significant draw-down of DIC during the assay might bias results. Higher label addition generally increases the resolution of the assay and lowers the consequences of offsets in the blank value. It should be noted, however, that high concentrations of ^14^C in spike solutions can affect not only the isotopic but also the chemical conditions in the cuvette (e.g., pH and DIC).

Overall, our sensitivity study revealed that the ^14^C disequilibrium method is a straightforward and robust assay, which is very useful for resolving the C_i_ source of phytoplankton over a range of different pH values. It is important to realize, however, the pH of assay buffers has the potential to significantly affect the C_i_ uptake behavior of cells.

## Conclusions

Our data clearly demonstrate that both life-cycle stages of *E. huxleyi* predominantly use CO_2_ as C_i_ source for photosynthesis under typical present-day and future CO_2_ levels, but constitutively express HCO_3_^−^ transporters allowing them to directly use HCO_3_^−^ when CO_2_ becomes limiting. Under bloom conditions, where pH values can easily increase to 8.5 or higher, cells might, therefore, be able to maintain efficient C_i_ acquisition. Future research needs to investigate whether and how the assay pH governs the mode of C_i_ acquisition also in other coccolithophores species or phytoplankton taxa and how this may alter the energy budget of cells. Results from previous studies may need re-consideration in the light of our data showing strong short-term pH effects on C_i_ uptake of phytoplankton.

## Electronic supplementary material

Below is the link to the electronic supplementary material.

Supplementary material 1 (XLSX 120 kb)
